# Fine Structures of 8-G-1-(*p*-YC_6_H_4_C ≡ CSe)C_10_H_6_ (G = H, Cl, and Br) in Crystals and Solutions: Ethynyl Influence and Y- and G-Dependences

**DOI:** 10.1155/2009/347359

**Published:** 2009-09-27

**Authors:** Satoko Hayashi, Kentaro Yamane, Waro Nakanishi

**Affiliations:** Department of Material Science and Chemistry, Faculty of Systems Engineering, Wakayama University, 930 Sakaedani, Wakayama 640-8510, Japan

## Abstract

Fine structures of 8-G-1-(*p*-YC_6_H_4_C ≡ CSe)C_10_H_6_ [**1** (G = H) and **2** (G = Cl): Y = H (**a**), OMe (**b**), Me (**c**), F (**d**), Cl (**e**), CN (**f**), and NO_2_ (**g**)] are determined by the X-ray analysis. Structures of **1**, **2**, and **3** (G = Br) are called **A** if each Se–C_sp_ bond is perpendicular to the naphthyl plane, whereas they are **B** when the bond is placed on the plane. Structures are observed as **A** for **1a**–**c** bearing Y of nonacceptors, whereas they are **B** for **1e**–**g** with Y of strong acceptors. The change in the structures of **1e**–**g** versus those of **1a**–**c** is called Y-dependence in **1**. The Y-dependence is very specific in **1** relative to 1-(*p*-YC_6_H_4_Se)C_10_H_7_ (**4**) due to the ethynyl group: the Y-dependence in **1** is almost inverse to the case of **4** due to the ethynyl group. We call the specific effect “*Ethynyl Influence.*” Structures of **2** are observed as **B**: the **A**-type structure of **1b** changes dramatically to **B** of **2b** by G = Cl at the 8-position, which is called G-dependence. The structures of **2** and **3** are examined in solutions based on the NMR parameters.

## 1. Introduction

**Scheme 1:**
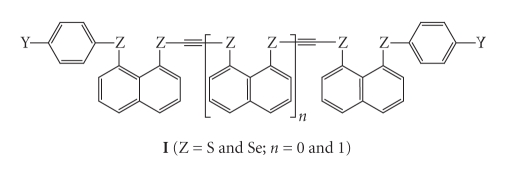


We are much interested in extended hypervalent bonds [*m* center–*n* electron bonds (*mc* − *ne* : *m* ≥ 4) [1–11] higher than 3c–4e [1, 12–14]. The nature of 4c–6e [1–5] is demonstrated to be very different from that of 3c–4e [1, 12–18]. Our strategy to construct the extended hypervalent bonds is to employ the interactions caused by direct orbital overlaps between nonbonded atoms [1–11, 19–21]. Weak interactions control fine structures and create delicate functionalities of materials [15, 22–40]. Recently, extended hypervalent bonds are shown to play an important role in physical, chemical and biological properties of the compounds [41–50]. On the other hand, the ethynyl group and the derivatives are of great importance as building blocks in the material design of high functionality [51–64]. Indeed, the ethynyl *π* and π* orbitals play an important role to appear specific properties in 8-G-1-(Ph_3_SiC ≡ C)C_10_H_6_ (G = OMe and NMe_2_) [65], but the ethynyl *σ* and σ* orbitals must also be of interest to originate the functionalities of materials. It will be great interest if the ethynyl group is joined to the extended hypervalent bonds constructed by the group 16 elements, such as **I** (Scheme 1).

**Scheme 2:**
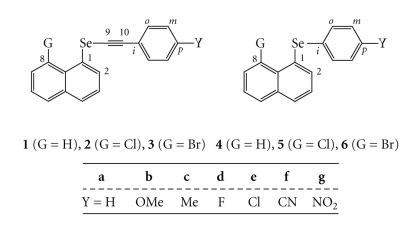


As the first step to clarify the factors to control the fine structures of the ethynyl joined extended hypervalent compounds such as **I** ^1^, the ethynyl influence and Y- and G-dependences as the factors to control fine structures of 8-G-1-(*p*-YC_6_H_4_C ≡ CSe)C_10_H_6_ [**1** (G = H) [66], **2** (G = Cl) [67], and **3** (G = Br) [67]: Y = H (**a**), OMe (**b**), Me (**c**), F (**d**), Cl (**e**), CN (**f**), and NO_2_ (**g**)] are elucidated (Scheme 2). The Y- and G-dependences are also discussed for 8-G-1-(*p*-YC_6_H_4_Se)C_10_H_6_ [**4** (G = H) [22], **5** (G = Cl) [16], and **6** (G = Br) [16] for convenience of comparison.** 1**–**3** are prepared and the structures of some compounds are determined by the X-ray crystallographic analysis.

**Scheme 3:**
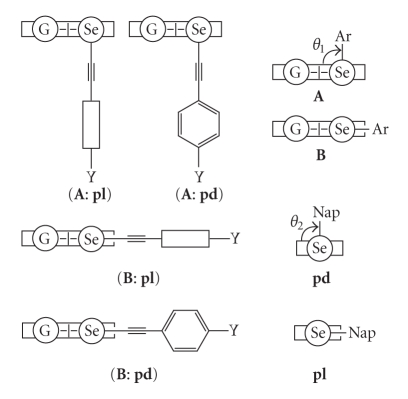
Plausible structures of **1**–**3**: **A** and **B** notation for naphthyl group, **pd** and **pl** for phenyl group, and the combined one for **1**–**3**.

Structures of the naphthalene system are well explained by the three types, **A**, **B**, and **C**, in our definition, where the Se–C_sp_ bond is perpendicular to the naphthyl plane in **A**, it is placed on the plane in **B**, and **C** is intermediate between **A** and **B** [4, 15, 16, 22, 23, 68]. The **A**, **B**, and **C** notations are employed for the structures around the Se–C_Nap_ bonds in **1**–**6**. The planar (**pl**) and perpendicular (**pd**) notations are also used to specify the structures of **1**–**6**, where they specify the conformers around the Se–C ≡ C–C_Ar_ (abbreviated Se–C_Ar_) bonds in **1**–**3** and those around Se–C_Ar_ in **4**–**6**. Scheme 3  illustrates plausible structures of **1**–**3**. Combined notations such as (**A**: **pl**) and (**B**: **pd**) are employed for the structures. The structures of **4** are **B** for Y of donating groups such as OMe, whereas they are **A** for Y of accepting groups such as NO_2_ [22]. We call the results Y-dependence. The magnitude of the p(Se)–*π*(Ar/Nap) conjugation must be the origin of Y-dependence in **4**.

 Here, we report the fine structures of **1** and **2** determined by the X-ray crystallographic analysis as a factor to control the fine structures. We call the factor “*Ethynyl Influence*” in **1** and the G-dependence arise from the nonbonded n_p_(G) ⋯ σ*(Se–C_sp_) 3c–4e interaction or the G ⋯ Se–C_sp_–C_sp_–C_sp^2^_ 5c–6e type interaction in **2** and **3**. The behaviors of **1**–**3** in solutions are also examined, containing the selective ^1^H, ^13^C-NOE difference spectroscopic measurements, to estimate the efficiency of the factors based on NMR parameters.

## 2. Experimental

### 2.1. Materials and Measurements

Manipulations were performed under an argon atmosphere with standard vacuum-line techniques. Glassware was dried at 130°C overnight. Solvents and reagents were purified by standard procedures as necessary. 

Melting points were measured with a Yanaco-MP apparatus of uncollected. Flash column chromatography was performed on silica gel (Fujisilysia PSQ-100B), acidic and basic alumina (E. Merck). **1**–**3** were prepared by the methods described elsewhere [67, 68].

 NMR spectra were recorded at 297 K on a JEOL AL-300 MHz spectrometer (^1^H, 300 MHz; ^77^Se, 57 MHz) on a JEOL ECP-400 MHz spectrometer (^1^H, 400 MHz; ^13^C, 100 MHz) in chloroform-*d* solutions (0.050 M)^2^. Chemical shifts are given in ppm relative to one of TMS for ^1^H NMR spectra and relative to reference compound Me_2_Se for ^77^Se NMR spectra.

### 2.2. X-Ray Crystal Structure Determination

Single crystals of some of **1** and **2** were obtained by slow evaporation of dichloromethane-hexane and/or ethyl acetate solutions at room temperature. X-ray diffraction data were collected on a Rigaku/MSC Mercury CCD diffractometer equipped with a graphite-monochromated MoK*α* radiation source (*λ* = 0.71070 Å) at 103(2) K. The structures were solved by direct methods (SIR97) [69] for **1a**–**c**, **1e**–**g**, and** 2e** and (SHELXS-97) [70] for **2b**, and (SIR2004) [71] for **2g** and refined by the full-matrix least squares method on *F* ^2^ for all reflections (SHELXL-97) [72]. All of the nonhydrogen atoms were refined anisotropically. CCDC-666789 (**1a**), CCDC-666790 (**1b**), CCDC-666791 (**1c**), CCDC-666792 (**1e**), CCDC-666793 (**1f**), CCDC-666794 (**1g**), CCDC-687206 (**2b**), CCDC-687207 (**2e**), and CCDC-687208 (**2g**) are available. These data can be obtained free of charge via http://www.ccdc.cam.ac.uk/conts/retrieving.html, or from the Cambridge Crystallographic Data Centre, 12 Union Road, Cambridge CB2 1EZ, UK; fax: (+44) 1223-336-033; or e-mail: deposit@ccdc.cam.ac.uk.

## 3. Results and Discussion

### 3.1. Structures of 1 and 2 in Crystals

**Figure 1:**
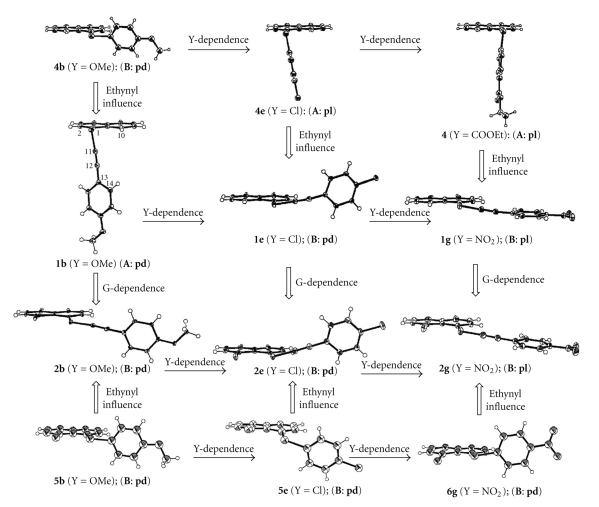
*Ethynyl Influence* in **1** and **2** and Y-dependence in **1**, **2**, and **4**–**6**.

Single crystals were obtained for **1a**–**c**, **1e**–**g**, **2b**, **2e**, and **2g** via slow evaporation of dichloromethane-hexane or ethyl acetate solutions. The X-ray crystallographic analyses were carried out for a suitable crystal of each compound. One type of structure corresponds to **1b**, **1c**, **1e**–**g**, **2b**, **2e**, and **2g** and two-type ones to **1a** in the crystals. The crystallographic data and the structures are reported elsewhere [66, 67]. Figure 1 summarizes structures of **1** and **2**, relative to **4**–**6**. Table 1 collects the selected interatomic distances, angles, and torsional angles, necessary for the discussion. The atomic numbering scheme is shown for **1b** in Figure 1, as an example.

As shown in Figure 1 and Table 1, the structure of **1** is **A** for Y of nonacceptors (**1 **(**A**)) such as H (**a**), OMe (**b**), and Me (**c**), whereas that of **1** is **B** for Y of acceptors (**1 **(**B**)) such as Cl (**e**), CN (**f**), and NO_2_ (**g**) (Scheme 2). The results are quite a contrast to the case of **4**, where the structure of **4** is **B** with Y = OMe, and they are **A** when Y = Cl and NO_2_. The ethynyl group interrupted between *p*-YC_6_H_4_ and Se changes the structures dramatically: (**B**: **pd**) of **4b** to (**A**: **pd**) of **1b**, (**A**: **pl**) of **4e** to (**B**: **pd**) of **1e**, and (**A**: **pl**) of **4** (Y = CO_2_Et) to (**B**: **pl**) of **1g**, where Y = CO_2_Et is employed in place of Y = NO_2_ for **4** [22]. The direction of Y-dependence in **1** is just the inverse to the case of **4**. We call the factor to determine the fine structure of **1** “*Ethynyl Influence*”.

The change in the structures of **1e**–**g** versus those of **1a**–**c** is called Y-dependence in **1**. The Y-dependence is very specific in **1** relative to **4** due to the ethynyl group: the Y-dependence in **1** is almost inverse to the case of **4** due to the ethynyl group. We call the specific effect “*Ethynyl Influence.*”

In the case of the structures of **2**, they are (**B**: **pd**) for **2b** and **2e** and (**B**: **pl**) for **2g**. The results exhibit that **1b** (**A**: **pd**) changes dramatically to **2b** (**B**: **pd**) by G = Cl at the 8-position in **2**. We call the effect G-dependence in **2**. The effect fixes the structure of **2** to **B**. While the variety of structures such as (**A**: **pd**), (**B**: **pd**), and (**B**: **pl**) are observed in **1**, the observed structure is only **B** in **2**. The observation is quite different from that in **1**, again. The observed structure of **1g** is substantially different from that of **6g**. Y-dependence in **2** must be very similar to that in **1**.

 After explanation of the observed structures of **1** in crystals, the role of crystal packing forces is examined in relation to the fine structures of **1**.

### 3.2. Crystal Packing Forces as Factor to Determine Fine Structure of 1

**Figure 2:**
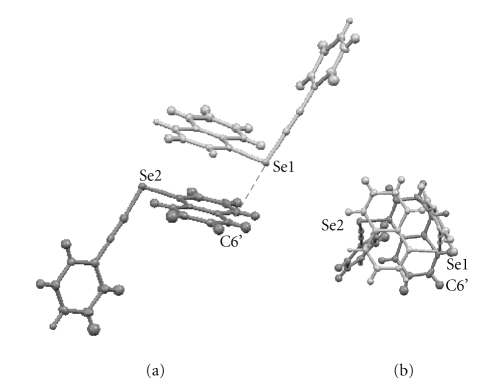
Dimer formed in **1a**, which contains **1a**_*A*_ and **1a**_*B*_: (a) a side view (*r*(Se1—C6′): 3.392 Å) and (b) a top view.

The structures of **1a**–**c** are observed as dimers. Figure 2 shows the dimer formed from **1a**, which contains **1a**_*A*_ and **1a**_*B*_. Se atoms in the **1a** dimer are in short contact with C at the 6′ position of the partner molecule, and the Se1—C6′ distance is 3.392 Å. Dimers of **1b** and **1c** are essentially the same as that of **1a**. An Se atom in the **1b** dimer is in short contact with C at the 4′ position of the partner molecule. The overlap between two naphthyl planes seems larger for the **1b** dimer relative to the **1a** dimer. The driving force of the dimer formation must be the energy lowering effect by the *π*-stacking of the naphthyl groups. The *π*(C) ⋯ σ*(Se–C_sp_) 3c–4e interaction must also contribute to stabilize the dimers. The dimer formation must stabilize the **A** structure for **1a**–**c**. It would be difficult to conclude whether the structures are **A** or **B** without such dimer formation. However, the **A** structure of **1a**–**c** would be suggested without the aid of the dimer formation by considering the electron affinity of naphthalene (NapH) and the evaluated values for *p*-YC_6_H_4_CCH (Y = H, OMe, and Me), which are the components of **1**.

After the establishment of the structures of **1** and **2** in crystals, next extension is to examine the structures of **1**–**3** in solutions.

### 3.3. Behavior of **1**–**3** in Solutions Based on NMR Spectroscopy

9-(Arylselanyl)anthracenes [9-(*p*-YC_6_H_4_Se)C_14_H_9_: **7**] and 1-(arylselanyl)anthraquinones [9-(*p*-YC_6_H_4_Se)C_14_H_7_O_2_: **8**] with all Y shown in Scheme 2  are demonstrated to serve as the standards for the structures of (**A**: **pl**) and (**B**: **pd**) in solutions, respectively [73, 74]. Scheme 3  illustrates the structures of **7 **(**A**: **pl**) and **8 **(**B**: **pd**). Consequently, ^1^H and ^77^Se NMR chemical shifts of **1**–**3** are also served as the standards to determine the (**A**: **pl**) and (**B**: **pd**) structures in solutions. The structures and the behaviors of **1**–**3** are investigated in solutions based on the NMR chemical shifts of **1**–**3** by comparing those of **7 **(**A**: **pl**) and **8 **(**B**: **pd**).

^1^H and ^77^Se NMR chemical shifts of **1**–**3** were measured in chloroform-*d* solutions (0.050 M) at 297 K^2^. Table 2 collects the substituent induced *δδ*(H_2_), *δ*(H_8_), and *δ*(Se) values for **1**–**3**. Table 2 also collects the values for **7 **(**A**: **pl**) and **8 **(**B**: **pd**). The values of **1**–**3 **change depending on Y, although the magnitudes are not so large. How are the changes in the chemical shifts depending on Y correlated to the structural changes in solutions? The changes in **1**–**3** are examined by comparing those in **7 **(**A**: **pl**) and **8 **(**B**: **pd**).

To organize the process for the analysis, *δ*(H_2_: **3**) and *δ*(Se: **3**) are plotted versus *δ*(H_2_: **2**) and *δ*(Se: **2**), respectively. Figure 2 shows the plots. The correlations are given in Table 3 (entries 1 and 2, resp.). The correlations are very good (*r* ≥ 0.995). The results show that the structure of each member in **3** is very close to that of **2**, in solutions. Therefore, the structures of **2** should be analyzed from the viewpoint of the orientational effect, together with those of **1**. The anisotropic effect of the C ≡ C bond in **3** (G = Br) might be stronger than that in **2 **(G = Cl), since *δ*(H_2_) values of **3** (8.39–8.44) are observed slightly more downfield than those of **2** (8.30–8.38). 

**Figure 4:**
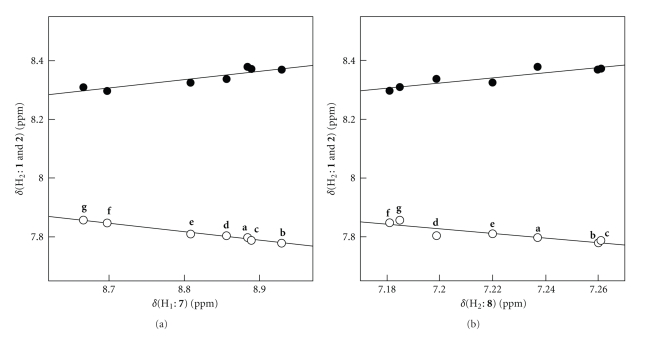
Plots of *δ*(H_2_: **1** and **2**) versus *δ*(H_1_: **7**) (a) and *δ*(H_2_: **8**) (b): ○ for **1** and 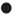 for **2**.

 As shown in Table 2, the *δ*(H_2_) values of **7 **(**A**: **pl**) and **8 **(**B**: **pd**) appear at 8.67–8.93 and 7.18–7.26, respectively, which should be the anisotropic effect of the phenyl group: the H_2_ atom in **7 **(**A**: **pl**) exists on the in-plane area of the phenyl group, whereas it resides upside of the group in **8 **(**B**: **pd**). On the other hand, *δ*(H_2_) of **1 **appear at 7.78–7.86, whereas those of **2** are 8.30–8.38. We must be careful when the structures of **1**–**3** are considered based on *δ*(H_2_), since H atoms above the C ≡ C bond is more deshielded which is just the inverse anisotropic effect by the phenyl group. The magnitudes of the former must be smaller than of the latter. Namely, the structures of **2** and **3** are expected to be **B** in solutions, although the slight equilibrium between **A** and **B** could not be neglected. The structures of **1** would be **A** in solutions, although **A** may equilibrate with **B** to some extent. Figure 4 shows the plots of *δ*(H_2_: **1** and **2**) versus *δ*(H_1_: **7**) and *δ*(H_2_: **8**). The plots appear from downfield to upfield in an order of *δ*(H_2_: **1**) ≪ *δ*(H_2_: **2**). The correlations are given in Table 3 (entries 3–6), which support above discussion.

**Figure 5:**
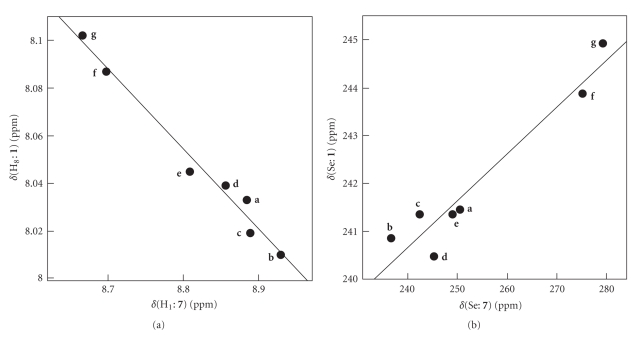
Plots of *δ*(H_8_: **1**) versus *δ*(H_1_: **7**) (a) and *δ*(Se: **1**) versus *δ*(Se: **7**) (b).

*δ*(H_8_: **1**) and *δ*(Se: **1**) are plotted versus *δ*(H_1_: **7**) and *δ*(Se: **7**), respectively. Figure 5 shows the results. The correlations are shown in Table 3 (entries 7 and 8, resp.). The correlation of the former is good, which means that (**A**: **pl**) contributes predominantly to the structures of **1**, although the correlation constant is a negative value of –0.34. The negative value would be the reflection of the inverse anisotropic effect between the phenyl *π* system and the ethynyl group. It is concluded that the structures of **1** in solutions are substantially (**A**: **pl**) with some contributions of (**B**: **pd**) and/or (**B**: **pl**) through the equilibrium. The correlation for the plot of *δ*(Se: **1**) versus *δ*(Se: **7**) also supports the conclusion, although **A** is suggested to equilibrate with **B** in solutions.

**Scheme 5:**
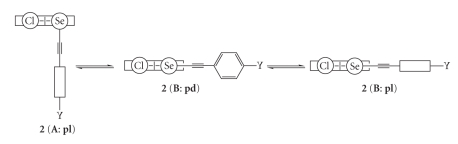
Equilibrium in **2**: **2** (**B**: **pd**) is expected to be the substantial structure in solutions.

Indeed, the preferential contribution of **B** is predicted for **2**, but, the plots of *δ*(H_2_: **2**) versus *δ*(H_2_: **8**) do not give good correlations (Panel (b) of Figure 4 and entry 6 in Table 3). Although not shown, the plot of *δ*(Se: **2**) versus *δ*(Se: **8**) did not give good correlation either (entry 10 in Table 3). The plots of *δ*(Se: **2**) versus *δ*(Se: **7**) gave rather good correlation (entry 9 in Table 3). The discrepancy must come mainly from the equilibrium between (**B**: **pd**) and (**B**: **pl**). Namely, the structures of **2** are predominantly **B**, which are in equilibrium between (**B**: **pd**) and (**B**: **pl**) especially for Y of strong electron accepted groups (Scheme 5). The equilibrium with **A** would exist but the contribution must be small for most of Y.

 The **B** structures of **2** and **3** in solutions are determined based on the large downfield shifts of *δ*(H_2_: **2**) and *δ*(H_2_: **3**) versus *δ*(H_2_: **1**). The reason for the structural determination in solutions will be discussed, next.

### 3.4. Selective ^1^H, ^13^C -NOE Difference Spectroscopy for **1e** and **2e** in Solutions

**Figure 6:**
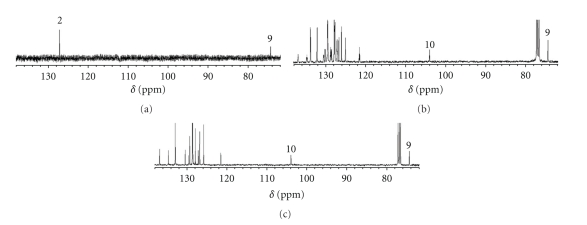
(a) Selective ^1^H, ^13^C-NOE difference spectrum, (b) off-resonance decoupling spectrum, and (c) completely ^1^H decoupled spectrum for **2e**.

**1e** (G = H, Y = Cl) and **2e** (G = Y = Cl) were employed for the selective ^1^H, ^13^C-NOE difference spectroscopy. ^13^C NMR spectra were measured for **1e** and **2e** under the completely ^1^H decoupling mode, the off-resonance decoupling mode, and the selective ^1^H, ^13^C -NOE difference mode at the *δ*(H_2_) frequency: The atom numbers are shown in Scheme 2. Figure 6 shows the ^13^C NMR spectra for **2e**. Panels (a)–(c) of Figure 6 correspond to the selective ^1^H, ^13^C -NOE difference spectroscopy, off-resonance decoupling spectroscopy, and completely decoupling spectroscopy, respectively. As shown in Panel (a) of Figure 6, the selective irradiation at the *δ*(H_2_) frequency of **2e** enhances exclusively the ^13^C NMR signals of C_2_ and C_9_ of **2e**, relative to others. On the other hand, only ^13^C NMR signal of C_2_ of **1e** is enhanced relative to others, when the *δ*(H_2_) frequency of **1e** is selectively irradiated, although not shown. The results must be the reflection of the expectation that H_2_ is very close to C_9_ in **2e** to arise the nuclear interaction resulting in the NOE enhancement, whereas such interaction does not appear between H_2_ and C_9_ in **1e** due to the long distance between them. Namely, structures of **1e** and **2e** are demonstrated to be **A** and **B**, respectively, in solutions on the basis of the homonuclear NOE difference spectroscopy. The structure of **3e** must also **B** in solutions on the analogy of the case in **2e**. The results strongly support above conclusion derived from the *δ*(H_2_) values of **1**–**3**.

## 4. Conclusions

The behavior of ethynylchalcogenyl groups is examined as the factor to control fine structures. Fine structures of 8-G-1-(*p*-YC_6_H_4_C ≡ CSe)C_10_H_6_ [**1** (G = H) and** 2** (G = Cl): Y = H (**a**), OMe (**b**), Me (**c**), F (**d**), Cl (**e**), CN (**f**), and NO_2_ (**g**)] are determined by the X-ray crystallographic analysis. Structures are (**A**: **pd**) or (**A**: **np**) for **1a**–**c** bearing Y of nonacceptors, it is (**B**: **pd**) for **1e** with Y = Cl, and they are (**B**: **pl**) for **1f** and **1g** having Y of strong acceptors of CN and NO_2_. The Y-dependence observed in **1** is just the opposite to the case of 1-(*p*-YC_6_H_4_Se)C_10_H_7_ (**4**). The factor to control the fine structures of **1** is called “*Ethynyl Influence.*” The structures are determined by the X-ray crystallographic analysis for **2b**, **2e**, and **2g**. The structures are all **B** around the Se–C_Nap_ bonds, in our definition. The structures around the Se–C_Ar_ bonds are **pd** for **2b** and **2e** and **pl** for **2g**. The **1b** (**A**: **pd**) structure with Y = OMe changes dramatically to **2b** (**B**: **pd**) by G = Cl at the 8-position in **2**. The effect is called G-dependence. The G-dependence must arise from the energy lowering effect of the n_p_(Cl) ⋯ σ*(Se–C_sp_) 3c–4e interaction. The n_p_(Cl)–*π*(Nap)–n_p_(Se)–*π*(C* ≡ *C) interaction may also contribute to stabilize the structure. The structures of **1**, **2**, and** 3** (G = Br) are also examined in solutions based on the NMR parameters for (**A**: **pl**) of 9-(arylselanyl)anthracenes (**7**) and (**B**: **pd**) of 1-(arylselanyl)anthraquinones (**8**). The results show that **2** and **3** behave very similarly in solutions, and the structures of **2** and **3** are predominantly **B** in solutions with some equilibrium between **pd** and **pl** for the aryl groups. The selective ^1^H, ^13^C-NOE difference spectroscopic measurements strongly support that the structures are **A** for **1** and **B** for **2** and **3** in solutions derived from the *δ*(H_2_) values of **1**–**3**.

## Figures and Tables

**Scheme 4:**
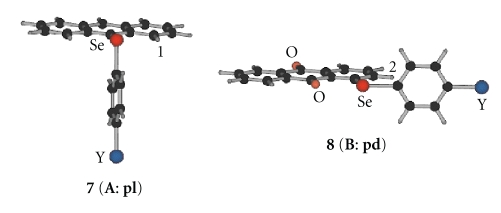
Illustration for the structures of **7 **(**A**: **pl**) and **8 **(**B**: **pd**).

**Figure 3:**
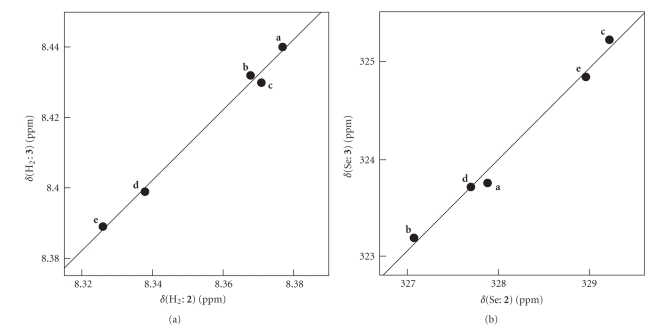
Plots of *δ*(H_2_: **3**) versus *δ*(H_2_: **2**) (a) and *δ*(Se: **3**) versus *δ*(Se: **2**) (b).

**Table 1:** Selected bond distances, angles, and torsional angles around Se atom, observed in **1** and **2** ^(a)^.

	**1a**_*A*_	**1a**_*B*_	**1b**	**1c**	**1e**	**1f**	**1g**
*r*(Se1, C1) (Å)	1.9321(19)1.9315(15)	1.9243(18)^(b) ^	1.936(2)	1.933(2)	1.935(2)	1.9335(19)	
*r*(Se1, C11) (Å)	1.8406(18)1.8233(15)	1.8381(19)^(c) ^	1.847(2)	1.844(2)	1.829(3)	1.825(2)	
∠C1Se1C11 (°)	98.07(7)	99.68(8)^(d)^	98.46(9)	99.07(10)	98.92(11)	98.93(9)	98.41(6)
∠C2C1Se1 (°)	117.37(14)122.17(11)	116.77(15)^(e) ^	117.61(16)	117.61(17)	121.26(19)	122.02(15)	
∠C10C1Se1 (°)	121.12(13)116.44(11)	121.74(14)^(f)^	120.89(15)	120.84(17)	116.86(18)	116.32(14)	
∠C10C1Se1C11 (°)	60.64(15)171.42(11)	62.36(15)^(g) ^	–79.69(17)	–78.92(19)	–169.24(19)	171.59(15)	
∠C1Se1C13C14 (°)	–54.80	–135.72^(h) ^	–58.78	126.88	–123.63	176.47	2.61
Structure	(**A**: **pd**)	(**A**: **np**)^(i)^	(**A**: **pd**)	(**A**: **pd**)	(**B**: **pd**)	(**B**: **pl**)	(**B**: **pl**)

	**2b**		**2e**		**2g**		

*r*(Se1, Cl1) (Å)	2.9495(16)		2.9753(17)		2.9454(15)		
*r*(Se1, C1) (Å)	1.946(4)		1.9458(18)		1.945(2)		
*r*(Se1, C11) (Å)	1.831(5)		1.8383(19)		1.829(2)		
*r*(Cl1, C9) (Å)	1.749(4)		1.7443(18)		1.744(2)		
∠Cl1Se1C11 (°)	179.18(15)		165.51(17)		175.70(15)		
∠Se1C11C12 (°)	167.2(4)		173.07(17)		170.6(2)		
∠C1Se1C11 (°)	99.7(2)		98.47(8)		99.32(10)		
∠C2C1Se1 (°)	118.0(3)		117.64(14)		117.70(17)		
∠C10C1Se1 (°)	121.7(3)		122.39(13)		122.21(16)		
∠C10C1Se1C11 (°)	170.5(3)		–164.16(15)		171.80(19)		
∠C1Se1C13C14 (°)	104.41(6)		102.59(8)		3.68(7)		
Structure	(**B**: **pd**)		(**B**: **pd**)		(**B**: **pl**)		

^(a) ^ The atomic numbering scheme is shown for **1b** in Figure 1, as an example. ^ (b)^ *r*(Se2, C19). ^ (c)^ *r*(Se2, C29). ^ (d) ^∠C19SeC29. ^ (e) ^∠C20C19Se2. ^ (f) ^∠C28C19Se2. ^ (g) ^∠C28C19Se2C29. ^ (h) ^∠C19Se2C31C32. ^ (i) ^ Intermediate structure between (**A**: **pd**) and (**A**: **pl**).

**Table 2:** ^1^H and ^77^Se NMR chemical shifts in **1**–**3**, together with those in **7** and **8** ^(a,b)^.

Y	OMe	Me	H	F	Cl	CN	NO_2_
	**b**	**c**	**a**	**d**	**e**	**f**	**g**
**1**							
*δ*(H_2_)	7.779	7.787	7.798	7.803	7.809	7.845	7.857
*δ*(H_8_)	8.010	8.019	8.033	8.038	8.045	8.087	8.102
*δ*(Se)	240.9	241.4	241.4	240.5	241.3	243.9	244.9
**2**							
*δ*(H_2_)	8.368	8.371	8.377	8.338	8.326	8.298	8.309
*δ*(Se)	327.1	329.2	327.9	327.7	329.0	332.7	334.2
**3**							
*δ*(H_2_)	8.432	8.430	8.440	8.399	8.389		
*δ*(Se)	323.1	325.2	323.7	323.7	324.8		
**7**							
*δ*(H_1_)	8.929	8.889	8.884	8.856	8.809	8.698	8.666
*δ*(Se)	236.8	242.4	249.0	245.4	247.5	275.2	279.3
**8**							
*δ*(H_2_)	7.260	7.261	7.237	7.199	7.220	7.181	7.185
*δ*(H_8_)	8.374	8.365	8.352	8.357	8.363	8.363	8.370
*δ*(Se)	497.3	503.4	512.3	502.2	505.3	504.1	509.8

^(a) ^ In CDCl_3_. ^ (b) ^ From TMS for *δ*(H) and from Me_2_Se for *δ*(Se).

**Table 3:** Correlations of *δ*(H) and *δ*(Se) in **1**–**3**, **7**, and **8** in solutions^(a)^.

Entries	Correlation	*a*	*b*	*r* ^2^	*n *(Y)
1	*δ*(H_2_: **3**) versus *δ*(H_2_: **2**)	0.994	0.11	0.996	5^(c)^
2	*δ*(Se:** 3**) versus *δ*(Se:** 2**)	0.939	16.1	0.995	5^(c)^
3	*δ*(H_2_: **1**) versus *δ*(H_1_: **7**)	–0.290	10.37	0.992	7^(b)^
4	*δ*(H_2_: **2**) versus *δ*(H_1_: **7**)	0.289	5.79	0.917	7^(b)^
5	*δ*(H_2_: **1**) versus *δ*(H_2_: **8**)	–0.784	13.47	0.895	7^(b)^
6	*δ*(H_2_: **2**) versus *δ*(H_2_: **8**)	0.856	2.16	0.906	7^(b)^
7	*δ*(H_8_: **1**) versus *δ*(H_1_: **7**)	–0.337	11.02	0.990	7^(b)^
8	*δ*(Se:** 1**) versus *δ*(Se:** 7**)	0.098	217.2	0.961	7^(b)^
9	*δ*(Se:** 2**) versus *δ*(Se:** 7**)	0.157	289.8	0.957	7^(b)^
10	*δ*(Se:** 2**) versus *δ*(Se:** 8**)	0.261	197.0	0.786	7^(b)^

^(a)^The constants (*a*, *b*, *r*) are defined by *y* = *ax* + *b* (*r*: correlation coefficient). ^(b)^ Y = OMe, Me, H, F, Cl, CN, and NO_2_. ^(c)^ Y = OMe, Me, H, F, and Cl.
